# Greater intramuscular adipose tissue of the quadriceps in older inpatients at post-acute hospital admission is more strongly related to a low rate of home discharge than a loss of muscle mass

**DOI:** 10.1038/s41598-023-37094-0

**Published:** 2023-06-20

**Authors:** Naoki Akazawa, Keita Funai, Toshikazu Hino, Ryota Tsuji, Wataru Tamura, Kimiyuki Tamura, Akemi Hioka, Hideki Moriyama

**Affiliations:** 1 Department of Physical Therapy, Faculty of Health and Welfare, Tokushima Bunri University, Tokushima, Tokushima, Japan; 2 Department of Rehabilitation, Kasei Tamura Hospital, Wakayama, Wakayama, Japan; 3 Life and Medical Sciences Area, Health Sciences Discipline, Kobe University, Kobe, Hyogo Japan

**Keywords:** Health care, Medical research

## Abstract

This study aimed to examine the relationships between intramuscular adipose tissue and muscle mass of the quadriceps at post-acute hospital admission and the low rate of home discharge. This prospective study included 389 inpatients aged ≥ 65 years. Patients were divided into two groups according to the destination: home discharge (n = 279) and no-home discharge (n = 110) groups. The primary outcome was hospital discharge destination (home discharge or not). Intramuscular adipose tissue and muscle mass of the quadriceps were assessed at post-acute hospital admission using echo intensity and muscle thickness on ultrasound images, respectively. Logistic regression analysis was used for determining whether quadriceps echo intensity is related to home discharge. Quadriceps echo intensity was significantly and independently associated with home discharge (odds ratio [per 1 SD increase] = 1.43, *p* = 0.045). Quadriceps thickness was not associated with home discharge (odds ratio [per 1 SD increase] = 1.00, *p* = 0.998). Our study indicates that greater intramuscular adipose tissue of the quadriceps in older inpatients at post-acute hospital admission is more strongly related to a low rate of home discharge than a loss of muscle mass.

## Introduction

The Position Statements of the Sarcopenia Definition and Outcomes Consortium (SDOC) suggested that muscle mass measured by dual-energy X-ray absorptiometry is not a good predictor of mobility limitations, falls, hip fractures, and mortality^1^. In addition, the European Working Group of Sarcopenia in Older People 2 (EWGSOP2) suggested the importance of assessing not only muscle mass but also intramuscular adipose tissue as muscle quality for sarcopenia diagnosis^2^. These suggestions of the SDOC and EWGSOP2 have indicated the possibility that muscle quality assessment is more important than muscle mass assessment for older inpatients.

Increased intramuscular adipose tissue of the quadriceps is more strongly associated with decreased muscle strength^3–5^ and decreased sit-up, sit-down^3,6^, gait abilities^4,7–9^, and swallowing function^10^ than loss of muscle mass. More recent studies^11,12^ reported that a greater intramuscular adipose tissue of the quadriceps in older inpatients at post-acute hospital admission is associated with a worse recovery of activities of daily living (ADL) than the loss of muscle mass. Based on these findings, we hypothesized that greater intramuscular adipose tissue of the quadriceps at post-acute hospital admission is more strongly associated with a low rate of home discharge than a loss of muscle mass in older inpatients. However, these relationships remain unclear. Considering home discharge is one of the main rehabilitation goals in a clinical setting, revealing these relationships is essential. Furthermore, knowing these relationships is important for predicting prognosis and developing effective approaches aimed at discharging home in older inpatients. This study aimed to examine whether greater intramuscular adipose tissue of the quadriceps at post-acute hospital admission is associated with a low rate of home discharge than a loss of muscle mass in older inpatients.

## Materials and methods

### Study design and participants

This prospective study included older inpatients who were referred to the Department of Rehabilitation at Kasei Tamura Hospital. This hospital has post-acute and convalescent rehabilitation wards. Patients aged < 65 years or who lacked data or died during hospital stay were excluded from the study. A total of 455 inpatients were recruited. Of these, 66 patients who were aged < 65 years (n = 33), had lack of necessary data (n = 18), or died during hospital stay (n = 15) were excluded. Consequently, 389 inpatients participated in this study. Patients were divided into two groups according to the destination: home discharge (n = 279) and no-home discharge (n = 110) groups. Rehabilitation therapy including physical therapy, occupational therapy, and speech and swallowing therapy was carried out for all participants during hospitalization^11,12^. This study was approved by the ethic committee of Tokushima Bunri University and was conducted in accordance with the principles of the Declaration of Helsinki. All participants or their guardians provided informed consent prior to the study.

A recent study^13^ reported that the rate of home discharge from the post-acute hospital in patients with sarcopenia was lower than that in patients without sarcopenia. In addition, the skeletal muscle mass index of the sarcopenia and non-sarcopenia groups in the study were 5.1 ± 0.9 kg/m^2^ and 6.7 ± 1.2 kg/m^2^, respectively, and the effect size (d) of the comparison between skeletal muscle mass index in the sarcopenia and non-sarcopenia groups was 1.51. We expected the same degree of effect size to be observed in intramuscular adipose tissue of the quadriceps between the home and no-home discharge groups in our study. With an effect size of 1.51, a statistical power of 0.99, and a significance level of *p* < 0.05, at least 18 participants were required in each group. The sample size calculation was conducted using G* Power version 3.1.9.2 (Heinrich-Heine-Universität Düsseldorf, Düsseldorf, Germany)^11,12^.

### Outcome measures

The primary outcome was hospital discharge destination (home discharge or not). We also measured the characteristics of the participants within 72 h of admission including, intramuscular adipose tissue and muscle mass of the quadriceps, disease, age, sex, height, body weight, body mass index (BMI), subcutaneous fat mass of the thigh, swallowing function, inflammation, nutritional status, comorbidities, number of medications, number of units of rehabilitation therapy (1 unit of rehabilitation therapy = 20 min), and ADL. The length of hospital stay (days) and days from onset disease were measured at discharge^11,12^. The length of hospital stay was assessed based on the hospitalization period at Kasei Tamura hospital^11,12^. The majority of older inpatients at our hospital were admitted from another acute-phase hospital. In these patients, the days from onset disease were assessed as the total length of stay in both hospitals^11,12^.

### Measurements of intramuscular adipose tissue and muscle mass of the quadriceps and subcutaneous fat mass of the thigh

Transverse ultrasound images were obtained using a B-mode ultrasound system (NanoMaxx; SonoSite Japan, Tokyo, Japan) with a linear-array probe (L25n/13–6 MHz; Nanomaxx; SonoSite Japan)^4,8–12,14,15^. The intramuscular adipose tissue and muscle mass of the rectus femoris and vastus intermedius of all participants were assessed based on the echo intensity and muscle thickness^4,8–12,14,15^. The validity of intramuscular adipose tissue and muscle mass measurements using ultrasound has been confirmed in recent studies using magnetic resonance imaging^16–19^. Images of the rectus femoris and vastus intermedius were obtained at 30% of the distance from the anterior superior iliac spine to the proximal end of the patella^4,8–12,14,15^. The participants lay in the supine position with their lower limbs relaxed, while a water-soluble transmission gel was applied to the skin surface of the thigh^4,8–12,14,15^. The probe was pressed perpendicularly and lightly against the skin to prevent muscle deformation^4,8–12,14,15^. All ultrasound images were recorded by the same investigator (physical therapist), who had sufficient training in echo intensity and muscle thickness measurements^4,8–12,14,15^. Echo intensity was measured in one region of interest in the rectus femoris and vastus intermedius; the region selected included the maximum possible regions, while avoiding the bone and surrounding fascia^4,8–12,14,15^. To standardize all echo intensity measurements, the gain status was normalized to the initial setting of the ultrasound system^4,8–12,14,15^. In addition, the image depth was uniformed at 60 mm for all echo intensity and muscle thickness measurements^4,8–12,14,15^. The rectus femoris thickness was determined as the distance between the superficial adipose tissue-muscle interface and the deep muscle-muscle interface^4,8–12,14,15^, and that of the vastus intermedius was determined as the distance between the superficial muscle-muscle interface and the bone-muscle interface^4,8–12,14,15^. Echo intensity and muscle thickness were measured using ImageJ 1.49 software (National Institutes of Health, Bethesda, MD, USA)^3–6,8–12,14,15^. Echo intensity was determined by performing a computer-assisted 8-bit gray-scale analysis, and the mean echo intensity of the regions of interest was expressed as a value from 0 (black) to 255 (white)^3–12,14,15^. Higher echo intensity indicates greater intramuscular adipose tissue.^20^.

The echo intensity of the quadriceps was calculated as the mean echo intensity of the rectus femoris and vastus intermedius^4,8–12,14,15^. The mean echo intensity of the right and left quadriceps was used in the analysis^4,8–12,14,15^. The sum of the thickness of the rectus femoris and vastus intermedius was used as a measure of quadriceps thickness^4,8–12,14,15^. The mean thickness of the right and left quadriceps was used in the analysis^4,8–12,14,15^. The methods used for measuring the echo intensity and muscle thickness of the rectus femoris and vastus intermedius in our study group have been reported to have high reliability (intraclass correlation coefficients [1.1] = 0.857–0.959)^15^. The subcutaneous fat mass of the thigh was assessed based on the subcutaneous fat thickness^4,8–12,14,15^. Subcutaneous fat thickness was determined as the distance between the dermis and adipose tissue interface and the muscle–adipose tissue interface^4,8–12,14,15^. The mean subcutaneous fat thickness of the right and left thigh was used in the analysis^4,8–12,14,15^.

### Measures of other characteristics

Swallowing function was assessed by a speech therapist using the Food Intake Level Scale (FILS)^21^. The FILS is a 10-point observer-rated scale, with higher values indicating better swallowing function. A doctor assessed the inflammatory status by analyzing C-reactive protein (CRP) concentration. Nutritional status was assessed by a registered dietitian using the Geriatric Nutritional Risk Index (GNRI)^22^. The GNRI was calculated using the following formula: GNRI = (14.89 × serum albumin [g/dL]) + (41.7 × body weight [kg]/ideal body weight)^22^. The ideal body weight was defined based on a BMI of 22.0 kg/m^2^^23^. If the body weight/ideal body weight was ≥ 1.0, the value was recorded as 1^22^. Comorbidities were evaluated by a doctor using the updated Charlson Comorbidity Index (UCCI)^24^. ADL were assessed by an occupational therapist using the Barthel Index (BI)^25^. The BI is widely used in clinical settings and includes ordinal assessment (0–100 points)^25^. Lower BI scores indicate poor ability to perform ADL.

### Statistical analysis

All statistical analyses were conducted using SPSS version 24 software (IBM SPSS Japan, Tokyo, Japan). Variables were assessed for normality using the Shapiro–Wilk test. Parametric data are reported as mean ± standard deviation, whereas nonparametric data are expressed as median (interquartile range [IQR]). Characteristics between the home and no-home discharge groups were compared using student's t-test, Mann − Whitney U test, or chi-square test. Logistic regression analysis (forced entry method) was used for determining whether quadriceps echo intensity is related to home discharge. Home and no-home discharge were corded as 0 and 1, respectively, as the dependent variable. Independent variables were quadriceps echo intensity and thickness, age, sex (male = 1, female = 0), the subcutaneous fat thickness of the thigh, disease (reference: stroke), days from onset disease, length of hospital stay, UCCI, and number of units of rehabilitation therapy. When observing 0.9 or higher in correlation coefficients between independent variables, we judged multicollinearity is present. The echo intensity is reportedly influenced by subcutaneous fat thickness^26^. Based on this finding, we included subcutaneous fat thickness of the thigh as an independent variable in the multiple and logistic regression analyses. A *p*-value of < 0.05 was considered to indicate statistical significance.

## Results

**Figure 1:**
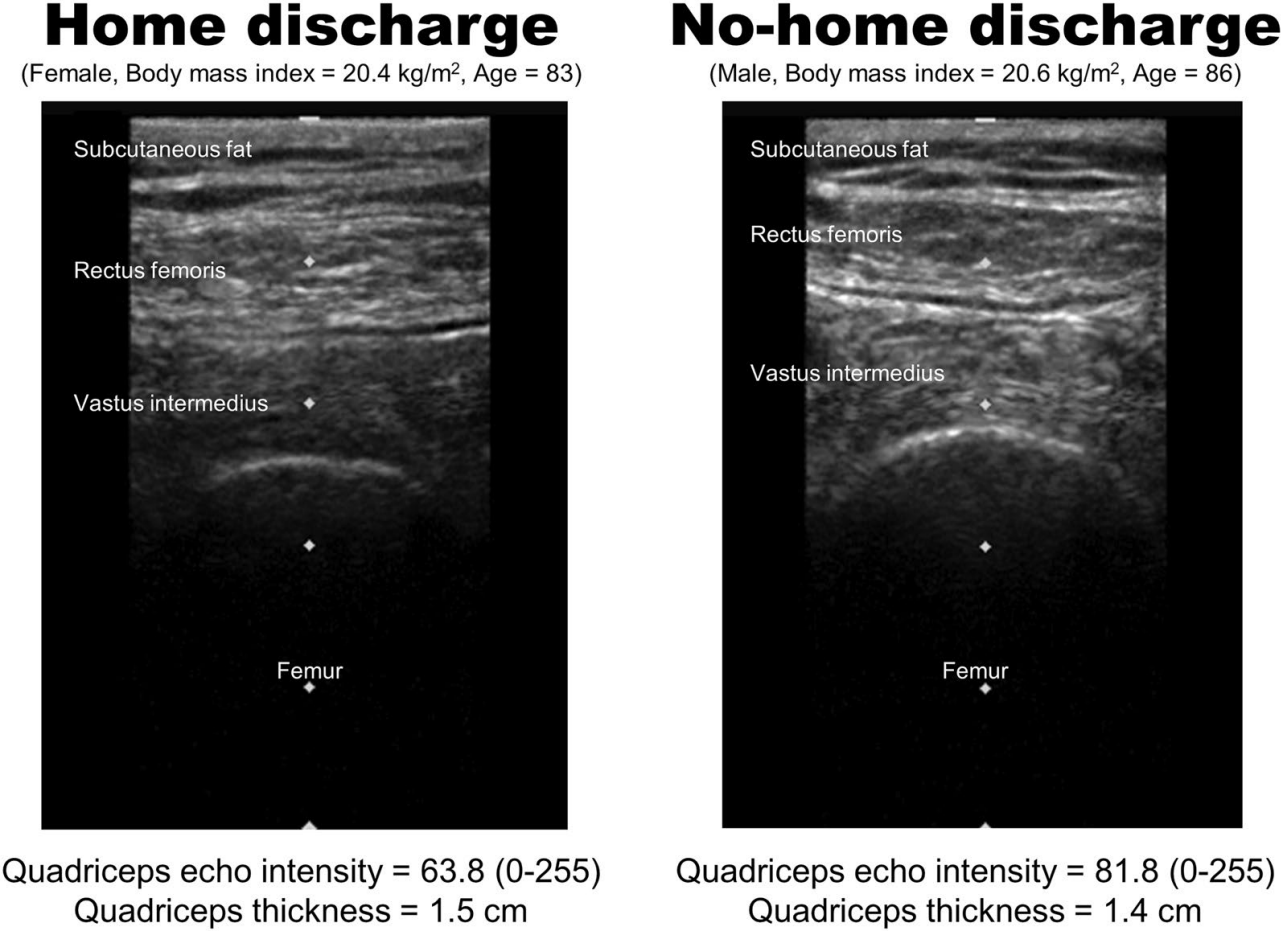
Typical ultrasound images of patients with home discharge and no-home discharge.

The rate of home discharge in this study was 71.7%. The median (IQR) of the age in the total participants was 83.0 (77.0–88.0) years. Diseases found among the participants were stroke (n = 59), fracture (n = 124, including hip fracture, compression fracture, pubic fracture, and other fracture), pneumonia (n = 63), and others (n = 143, including heart disease, kidney disease, chronic obstructive pulmonary disease, diabetes, cancer, dehydration, and urinary tract infection). Characteristics of participants and the results of the comparisons in characteristics between the home and no-home discharge groups are shown in Table 1. Quadriceps echo intensity, CRP, UCCI, days from onset disease, and length of hospital stay in the home discharge group were significantly lower than those of the no-home discharge group. Quadriceps thickness, BI scores at admission and discharge, BMI, FILS, GNRI, and albumin of the home discharge group were significantly higher than those of the no-home discharge group. No significant differences in other characteristics were observed between both groups. Figure 1 shows typical ultrasound images of patients with home discharge and no-home discharge.

**Table 1:** Characteristics of participants and comparisons of characteristics between the home and no-home discharge groups.

	Total	Home discharge group	No-home discharge group	*p*-value
	(n = 389)	(n = 279)	(n = 110)	
Age, years	83.0 (77.0–88.0)	82.0 (76.0–88.0)	83.5 (78.0–88.3)	0.151^a^
Sex, male/female	174 (44.7)/215 (55.3)	124 (44.4)/155 (55.6)	50 (45.5)/60 (54.5)	0.857^b^
Height, cm	151.0 (146.0–160.0)	151.0 (147.0–160.0)	152.5 (146.0–162.0)	0.778^a^
Body weight, kg	46.5 (39.7–54.0)	46.5 (40.1–54.5)	47.0 (38.0–52.3)	0.227^a^
Body mass index, kg/m^2^	20.0 (17.7–22.6)	20.3 (17.9–22.7)	19.2 (17.0–22.3)	0.042^a^
Length of hospital stay, days	58.0 (39.0–92.0)	55.0 (37.0–88.0)	77.0 (41.8–101.3)	0.003^a^
Days from onset disease	79.0 (49.0–111.0)	73.0 (47.0–105.0)	95.5 (61.8–132.0)	0.001^a^
Disease				0.098^b^
Stroke	59 (15.2)	39 (14.0)	20 (18.2)	
Fracture	124 (31.9)	98 (35.1)	26 (23.6)	
Pneumonia	63 (16.2)	40 (14.3)	23 (20.9)	
Others	143 (36.8)	102 (36.6)	41 (37.3)	
Quadriceps thickness, cm	1.2 ± 0.5	1.3 ± 0.5	1.1 ± 0.5	0.017^c^
Quadriceps echo intensity (gray-scale range, 0–255)	84.2 ± 21.6	82.0 ± 20.7	89.9 ± 22.8	0.001^c^
Subcutaneous fat thickness of the thigh, cm	0.4 (0.3–0.5)	0.4 (0.3–0.5)	0.4 (0.2–0.5)	0.100^a^
Food Intake Level Scale	8.0 (7.0–9.0)	8.0 (7.0–9.0)	7.0 (6.0–8.0)	< 0.001^a^
C-reactive protein, mg/dl	0.5 (0.4–1.7)	0.4 (0.4–1.2)	0.7 (0.4–2.7)	0.010^a^
Serum albumin, g/dl	3.4 (3.1–3.7)	3.5 (3.2–3.8)	3.2 (2.8–3.5)	< 0.001^a^
Geriatric nutritional risk index	87.9 (80.6–94.2)	89.0 (82.5–95.3)	83.7 (76.5–89.4)	< 0.001^a^
Updated Charlson comorbidity index	2.0 (0.0–3.0)	2.0 (0.0–3.0)	2.0 (1.8–4.0)	< 0.001^a^
Number of medications	7.0 (5.0–9.0)	7.0 (5.0–9.0)	7.0 (4.0–10.0)	0.436^a^
Number of rehabilitation therapy, units/day	3.0 (2.0–4.0)	3.0 (2.0–4.0)	3.0 (2.0–4.0)	0.361^a^
Barthel index score at admission	45.0 (20.0–60.0)	50.0 (30.0–60.0)	22.5 (5.0–45.0)	< 0.001^a^
Barthel index score at discharge	60.0 (40.0–80.0)	70.0 (50.0–85.0)	40.0 (15.0–55.0)	< 0.001^a^

Data are presented as median (interquartile range), n (%), or mean ± standard deviation.

^a^Mann − Whitney U test; ^b^Chi-square test; ^c^Student’s t-test.

The results of the logistic regression analysis for home discharge are shown in Table 2. Quadriceps echo intensity (odds ratio [per 1 SD increase] = 1.43, *p* = 0.045), days from onset disease (odds ratio = 1.01, *p* = 0.042), and UCCI (odds ratio = 1.16, *p* = 0.008) were significantly and independently associated with home discharge. Quadriceps thickness (odds ratio [per 1 SD increase] = 1.00, *p* = 0.998) was not associated with home discharge. The highest correlation coefficient among the independent variables was 0.746 (relationship between days from onset disease and length of hospital stay). The range of other correlation coefficients was − 0.722 to 0.372, and no multicollinearity was observed between the independent variables. The percentage of correct classifications of the logistic regression model was 74.6%.

**Table 2:** Logistic regression analysis for home discharge.

	B	SE	95% Confidence interval of OR	OR	*p*-value
Quadriceps echo intensity (per 1 SD increase)	0.36	0.18	1.01, 2.03	1.43	0.045
Quadriceps thickness (per 1 SD increase)	− 0.00	0.19	0.69, 1.45	1.00	0.998
Subcutaneous fat thickness of the thigh	0.62	0.63	0.54, 6.41	1.86	0.329
Age	0.02	0.02	0.99, 1.06	1.02	0.227
Sex	0.02	0.27	0.60, 1.72	1.02	0.954
Days from onset disease	0.01	0.01	1.00, 1.02	1.01	0.042
Length of hospital stay	− 0.00	0.01	0.99, 1.01	1.00	0.556
Updated Charlson comorbidity index	0.15	0.06	1.04, 1.29	1.16	0.008
Number of units of rehabilitation therapy	− 0.14	0.09	0.74, 1.04	0.87	0.121
Disease					
Stroke	(Reference)	–	–	–	–
Fracture	− 0.74	0.43	0.21, 1.10	0.48	0.081
Pneumonia	0.11	0.46	0.46, 2.73	1.12	0.807
Others	− 0.39	0.41	0.30, 1.51	0.68	0.339

B: Regression coefficient, SE: standard error, OR: odds ratio, SD: standard deviation.

## Discussion

This study examined the relationship between intramuscular adipose tissue of the quadriceps at post-acute hospital admission and home discharge in older inpatients. Our results indicate that greater intramuscular adipose tissue of the quadriceps in older inpatients at post-acute hospital admission is more strongly related to a low rate of home discharge than a loss of muscle mass.

Previous studies^13,27–29^ have reported that loss of muscle mass is related to a low rate of home discharge. This study is worthwhile in terms of indicating that assessing not only muscle mass but also intramuscular adipose tissue of the quadriceps and intramuscular adipose tissue is related to home discharge than muscle mass. Measurement of muscle mass does not always reflect the actual muscle mass because the area where muscle mass is measured includes both the muscle and intramuscular adipose tissue^30,31^, which potentially leads to the overestimation of the muscle mass^11,12,14^. These factors might have influenced our results (i.e., greater intramuscular adipose tissue of the quadriceps in older inpatients at post-acute hospital admission is more strongly related to a low rate of home discharge than a loss of muscle mass). Considering our results, assessing and intervening intramuscular adipose tissue is to be important for improving the rate of home discharge.

Based on our results, assessing intramuscular adipose tissue of the quadriceps in older inpatients may be important for predicting home discharge. EWGSOP2 has indicated the importance that assessing not only muscle mass but also muscle quality including intramuscular adipose tissue^2^. However, there is no consensus concerning muscle quality assessment, and EWGSOP2 has not indicated the cut-off value for muscle quality assessments in the diagnostic criteria for sarcopenia^2^. Therefore, a consistent agreement regarding muscle quality assessments must be formulated through further discussions.

Considering our results, improving intramuscular adipose tissue of the quadriceps may contribute to an increase in the rate of home discharge. Englund et al.^32^ has reported that physical activity and nutritional supplementation (whey protein and vitamin D) improved the intramuscular adipose tissue of the thigh in community-dwelling, mobility-limited older people. In addition, Kitajima et al.^33^ has confirmed that the presence of reduced intramuscular adipose tissue in the lumbar muscles of patients with liver cirrhosis whose serum albumin concentration was improved following supplementation with branched-chain amino acids. An intervention aimed at improving physical activity and nutritional status may be required to the improvement of intramuscular adipose tissue of the quadriceps in older inpatients.

The rate of home discharge from the post-acute hospital in a recent study^13^ that revealing sarcopenia negatively affects the rate of home discharge was 72.2% and this home discharge rate approximated our study (71.7%). Other recent studies^34–36^ have reported that the rates of home discharge from post-acute hospitals were 74.4 to 79.5%, and the rate of home discharge in this study was lower than these studies^34–36^. These may be attributed to differences in age of the participants between previous studies (mean age: 70.1–72.4 years)^34–36^ and this study (median age: 83.0 years). Considering them, suggested the rate of home discharge from the post-acute hospital in this study is not considered to be specific compared with that of previous studies^34–36^. In addition, an under 60 score in BI is considered a severe dependency condition^37^. The median (IQR) of the BI score at discharge in the no-home discharge group in this study was 40 (15.0–55.0). Considering almost all participants of the no-home discharge group were severe dependency conditions, the rate of home discharge in this study is considered to be valid.

A recent study^38^ reported that higher comorbidity in patients with total knee arthroplasty is related to the low rate of home discharge. The results of the logistic regression analysis in our study also indicated similar results. However, the relationship between intramuscular adipose tissue of the quadriceps at post-acute hospital admission and home discharge in older inpatients is not considered to depend on the comorbidity conditions, because this relationship was observed in adjusting for UCCI. In other words, greater intramuscular adipose tissue of the quadriceps in older inpatients at post-acute hospital admission may be related to a low rate of home discharge irrespective of higher or lower comorbidity conditions.

This study has some limitations. First, our results were obtained from a post-acute hospital. Therefore, whether a similar relationship is observed between intramuscular adipose tissue of the quadriceps and home discharge from an acute hospital remains unclear. Second, the number of units of rehabilitation therapy was adjusted in the logistic regression analysis in this study. However, we were not able to adjust the number of units of physical therapy, occupational therapy, and speech and swallowing therapy, respectively. In addition, physical function and sarcopenia were not examined. Therefore, the influences of these factors on the relationship between intramuscular adipose tissue of the quadriceps at post-acute hospital admission and home discharge in older inpatients remains unclear. Finally, the presence or absence of caregivers in a home may be related to home discharge^39^. However, we did not assess the presence or absence of caregivers in a home. Further study including this factor will be needed.

## Conclusions

Our study indicates that greater intramuscular adipose tissue of the quadriceps in older inpatients at post-acute hospital admission is more strongly related to a low rate of home discharge than a loss of muscle mass. Intramuscular adipose tissue of the quadriceps in older inpatients is considered to be a predictor for home discharge, and intervening for intramuscular adipose tissue may be important for improving the rate of home discharge.

## Supplementary Information

Supplementary Information.

## Data Availability

All data generated or analysed during this study are included in this published article and its supplementary information file.

## Abbreviations

SDOC: Sarcopenia Definition and Outcomes Consortium
EWGSOP2: European Working Group of Sarcopenia in Older People 2
ADL: Activities of daily living
BMI: Body mass index
FILS: Food Intake Level Scale
CRP: C-reactive protein
GNRI: Geriatric Nutritional Risk Index
UCCI: Updated Charlson comorbidity index
BI: Barthel Index
